# Identification of Three Small Molecules That Can Selectively Influence Cellular Manganese Levels in a Mouse Striatal Cell Model

**DOI:** 10.3390/molecules26041175

**Published:** 2021-02-22

**Authors:** Kyle J. Horning, Xueqi Tang, Morgan G. Thomas, Michael Aschner, Aaron B. Bowman

**Affiliations:** 1 Vanderbilt Brain Institute, Vanderbilt University, Nashville, TN 37232, USA; kyle.j.horning@gmail.com; 2 School of Health Sciences, Purdue University, West Lafayette, IN 47906, USA; tang484@purdue.edu (X.T.); thomasmg@purdue.edu (M.G.T.); 3 Department of Molecular Pharmacology, Albert Einstein College of Medicine Bronx, New York, NY 10461, USA

**Keywords:** manganese, small molecules, metal transport, divalent metals, neurotoxicity, metal homeostasis

## Abstract

Manganese (Mn) is a biologically essential metal, critical as a cofactor for numerous enzymes such a glutamine synthetase and kinases such as ataxia-telangiectasia mutated (ATM). Similar to other essential metals such as iron and zinc, proper levels of Mn need to be achieved while simultaneously being careful to avoid excess levels of Mn that can be neurotoxic. A lifetime of occupational exposure to Mn can often lead to a Parkinsonian condition, also known as “manganism”, characterized by impaired gait, muscle spasms, and tremors. Despite the importance of its regulation, the mechanisms underlying the transport and homeostasis of Mn are poorly understood. Rather than taking a protein or gene-targeted approach, our lab recently took a high-throughput-screening approach to identify 41 small molecules that could significantly increase or decrease intracellular Mn in a neuronal cell model. Here, we report characterization of these small molecules, which we refer to as the “Mn toolbox”. We adapted a Fura-2-based assay for measuring Mn concentration and for measuring relative concentrations of other divalent metals: nickel, copper, cobalt, and zinc. Of these 41 small molecules, we report here the identification of three that selectively influence cellular Mn but do not influence the other divalent metals tested. The patterns of activity across divalent metals and the discovery of Mn-selective small molecules has potential pharmacological and scientific utility.

## 1. Introduction

Several studies have shown a correlation between cellular manganese (Mn) homeostasis in the brain and risks for neurodegenerative diseases [1]. Under normal conditions, structures of the brain’s basal ganglia have relatively high levels of Mn, rendering these structures susceptible to Mn-induced neurotoxicity [2,3,4,5]. Occupational and environmental exposure to Mn increases risks for parkinsonism, and excessive and chronic exposure can lead to manganism, a neurological disease characterized by symptoms similar to Parkinson’s disease (PD) [6,7,8]. More recent research implicated a cellular deficit of Mn in the pathogenesis of Huntington’s disease (HD), a fatal neurodegenerative disease caused by expansion of a polyglutamine domain at the *N*-terminus of the huntingtin protein [9,10]. Altered Mn-responsive pathways and Mn-dependent biology are noted in the pathogenesis of both PD and HD, attesting to the essentiality of cellular Mn regulation in neuronal functions as well as the development of neurodegenerative diseases.

At a biological level, Mn functions as a cofactor for numerous kinases and several fundamental enzymes including glutamine synthetase, Mn superoxide dismutase, and arginase, whose deficiencies have been reported in HD and PD development. In HD, deficient arginase enzymatic activity in the urea cycle has been noted previously by our lab [10]. Reduced activity of Mn-dependent glutamine synthetase, mediator of the recycling of the glutamate neurotransmitter, has also been noted in HD [11,12]. These deficiencies occur in (but are not limited to) the striatum, where Mn selectively accumulates over time, representing a particularly vulnerable brain region in HD [13]. Parkin protein, encoded by *PARK2*, a gene linked to PD pathogenesis, has been shown to accumulate in Mn-treated dopaminergic cells. Genetic risk factors for early onset PD alter cellular susceptibility to Mn toxicity [14,15,16,17,18,19,20,21]. At excessive levels, kinase activities of vital cellular signaling cascades, including autophagy and insulin signaling, are responsive to increased Mn bioavailability and linked to HD pathology [22,23].

These findings corroborate the relatedness of active regulation of Mn transport and distribution and risk factors of neurodegeneration. Much like other biological trace metals, sufficient Mn needs to be achieved while avoiding toxic amounts. However, unlike other essential metals, the mechanisms that maintain Mn homeostasis have yet to be fully understood. Most of the known transporters involved in regulating Mn transport into and within cells (including neurons and glia) are non-selective, and also transport other essential metals. The known Mn transporters cannot be invoked to fully explain how intracellular Mn concentrations are selectively maintained without simultaneously and strongly influencing the concentrations of other divalent metals [24]. Aside from a few notable exceptions (e.g., divalent metal transporter 1 (DMT1) [25,26,27], transferrin receptor (TfR) [28,29], and solute carrier family 30 member 10 (SLC30A10) [30,31,32]), the manner by which Mn transporters regulate uptake and efflux of Mn versus the subcellular distribution of Mn is not well established. Mn is the only essential metal for which cellular homeostatic regulations are not clearly defined, despite numerous Mn-dependent enzymes throughout the cell. Since it has become likely that altered bioavailability of Mn contributes to cellular pathologies, it is reasonable to question if that could be a target for therapeutic treatments. Although some iron transporters are capable of trafficking Mn with some affinity, without Mn specificity they alone are insufficient to explain how cells maintain a homeostatic balance of Mn under normal conditions [24]. Understanding how cells maintain appropriate Mn concentrations in the brain under normal conditions is vital to understanding how a Mn deficit may occur in HD. Thus, we have taken an exploratory comprehensive approach to identify and characterize molecular tools that modify cellular Mn levels and potentially target mechanisms of brain Mn homeostasis.

Research to generate novel chemical tools to probe Mn transport mechanisms was performed previously by our lab using a high throughput screening approach [33]. The Fura-2-based fluorescent assay is referred to as the Cellular Fura-2 Manganese Extraction Assay, or CFMEA [34,35]. In brief, cells are exposed to extracellular Mn and incubated at physiological conditions before washing away the excess extracellular Mn in calcium-free buffer and using detergent to lyse open the cells. The released Mn is detected by the added fluorophore, Fura-2, and its fluorescence is quenched in a quantifiable, concentration-dependent matter at the calcium isosbestic point (360_Ex_/535_Em_) so that cellular calcium co-released with the Mn does not influence the fluorescence signal. While it is possible other endogenous divalent metals that alter Fura-2 fluorescence could interfere with Mn determination, the CFMEA was previously validated for accuracy of Mn quantification in the absence of elevated exposures to other divalent metals beyond what is normally found in cell culture media [34,35]. This is likely due to the fact that normal cellular levels of other divalent metals (aside from calcium) are too low to alter Fura-2 signals. This assay was subsequently optimized for a high-throughput screening format [36], and then subsequently used to screen ~40,000 small molecules across a diverse set of libraries [33]. The libraries screened include the National Institutes of Health (NIH) Clinical Collection I/II, Microsource Spectrum Collection, three different kinase inhibitor libraries, a bioactive lipid library, a non-steroidal anti-inflammatory library, as well as the ChemBridge and ChemDiv libraries; more details of the small molecule libraries screened and all compound structures examined in this paper are available in the original publication of the screen [33].

A total of 41 small molecules were identified to be capable of significantly increasing or decreasing intracellular Mn content in a concentration-dependent manner using a mouse striatal neural cell line (STH*dh*^Q7/Q7^) and our high-throughput CFMEA method [33,34,35,36]. This set of small molecules was coined the “Mn toolbox”, as each molecule may be used as a chemical tool to investigate Mn homeostasis in neuronal cells. Of the original 41 molecules previously identified, 37 are presently available and were examined in this study. We have previously validated in vivo activity for two molecules of the Mn toolbox (VU0063088 and VU0026921), that were shown to modulate Mn levels in *C. elegans*, through mechanisms independent of the species DMT-2 homologue, and induce protection from Mn neurotoxicity [37]. Likewise, VU0026921 was also found to be a modifier of metal homeostasis in gram-positive bacteria [38]. Here, we set out to test the hypothesis that cell-level homeostatic processes selectively regulate intracellular Mn to avoid hazardous concentrations in the brain, and thus small molecules could be found that specifically disrupt these processes as compared to other Mn-altering small molecules that may show affinity for other divalent metals. Understanding how cells selectively control intracellular Mn to avoid an excess or deficit is critical. When these processes are impaired, as they appear to be in PD and HD, our understanding of Mn homeostasis can illuminate therapeutic solutions to these devastating diseases.

Though the small molecules within the Mn toolbox were identified by our lab in 2014 [33], and have been confirmed to increase or decrease Mn accumulation in STH*dh*^Q7/Q7^ cells, their selectivity towards influencing Mn over other divalent transition metals has not been defined. Here using a modified version of the CFMEA [34] we find that of the set of small molecules tested, three appear to be Mn-selective. The patterns of activity across different divalent metals also may reveal insight into other regulatory mechanisms for metal homeostasis.

## 2. Results

### 2.1. Small Molecule Functional Stability and Effectiveness in Buffer

**Figure 1:**
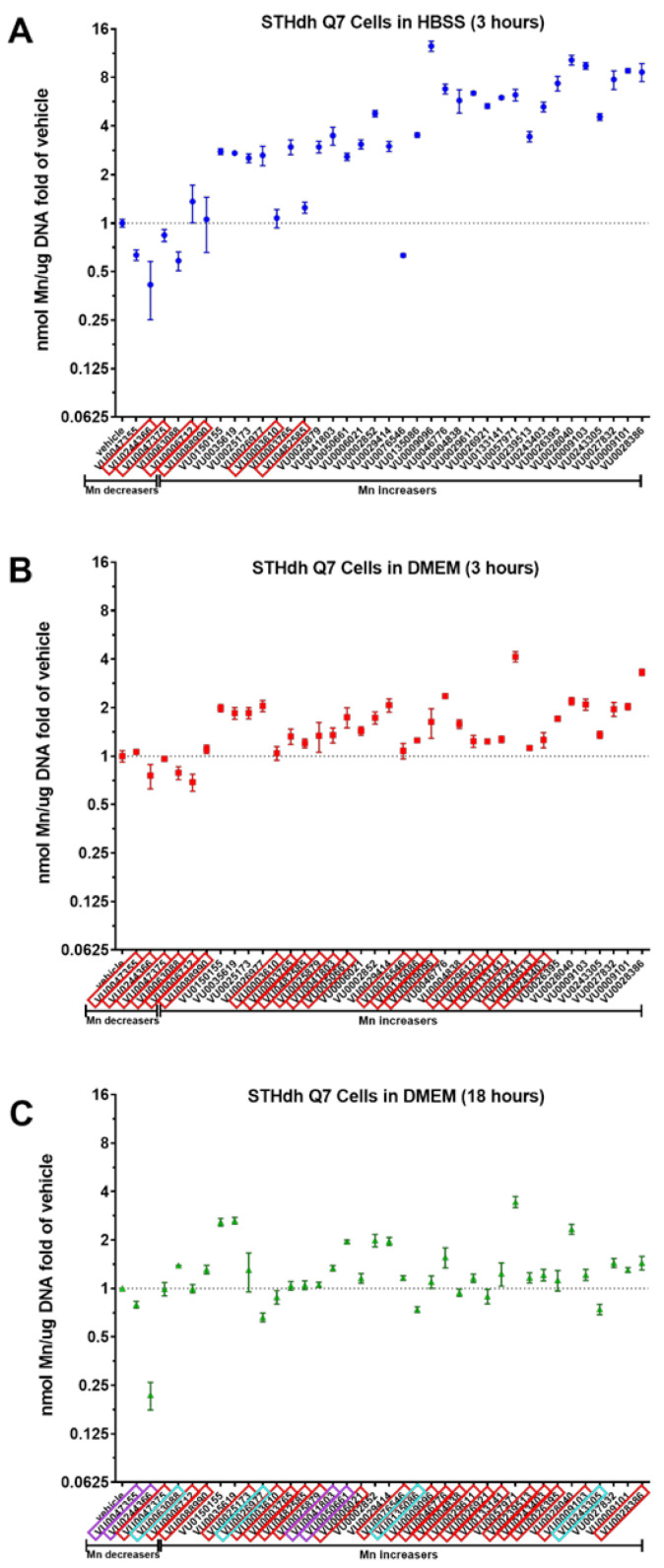
Small molecule modifiers of manganese have a tendency to lose their efficacy when exposed in culture media. A set of 37 small molecules identified to influence intracellular Mn in a high throughput screen (see reference 33), commonly referred to as the “Mn Toolbox”, were confirmed independently (**A**). A red box around each small molecule, identified using its assigned VU identity number (see reference 33), denotes non-significance as determined by Brown–Forsythe ANOVA and Welch’s ANOVA tests with Dunnett’s T3 multiple comparisons. The number of compounds that did not reach statistical significance increases when exposed in DMEM rather than HBSS (**B**). When incubated in media for 18 h (**C**), several small molecules had an impact on Mn when previously no statistically significant impact existed at 3 h (boxed in purple). Several small molecules changed directions (Mn-increaser to decreaser, or decreaser to increaser), which are denoted by a box in blue. Error bars represent standard deviation of three biological replicates.

For an optimally-controlled environment, the Mn toolbox was previously validated in Hanks balanced buffer solution (HBSS), but the question remained if the small molecules could work to influence cellular Mn uptake while the cells were in normal culture medium. Cells in culture medium are in a more complete extracellular environment, and can continuously be cultured long-term in their media, which is not viable in HBSS. Therefore, we tested the toolbox in the cells’ normal medium (10% FBS, 1% penicillin/streptomycin, in high glucose DMEM; see methods for further details). As a control, and in an attempt to confirm the results of the original screen, the toolbox was run in their standard conditions (co-incubation with 125 μM Mn in HBSS for 3 h at 33 °C), seen in Figure 1A. Brown–Forsythe ANOVA and Welch’s ANOVA tests were performed to confirm whether a small molecule significantly increased or decreased Mn uptake in cells compared to that of vehicle (DMSO). Small molecules boxed in red were molecules that were not confirmed by Dunnett’s T3 multiple comparison test to be significantly different from vehicle, with an adjusted *p* value less than 0.1. A total of six small molecules were not confirmed under the standard condition (Figure 1A). Three of these were previously found to decrease Mn uptake in the Q7 cells, while the other three were previously identified as Mn-increasers (i.e., molecules that lead to a net increase in total cellular Mn). Two Mn-decreasers (i.e., molecules that lead to a net decrease in total cellular Mn) and 28 Mn-increasers were confirmed in total.

A comparison of the entire toolbox in HBSS (Figure 1A) to the toolbox in media (Figure 1B), with a two-way ANOVA, revealed a clear main effect of the buffer used (*p* < 0.0001), comprising 26% of the variation, with an equally significant interaction between buffer and the small molecule, comprising 28% of the total variation. There was an overall decrease in effectiveness of the small molecules (decrease in fold change) when used in DMEM: an average 2.6-fold loss was measured. Accordingly, there were an additional two Mn-decreasers and twelve Mn-increasers that were not significantly different from vehicle when measured using Brown–Forsythe ANOVA and Welch’s ANOVA tests with Dunnett’s T3 multiple comparisons. These small molecules were also boxed in red (Figure 1B). What remained were zero Mn-decreasers and seventeen Mn-increasers that were capable of influencing Mn uptake while in media.

To test if any of these small molecules have Mn-altering activity during prolonged periods, the entire toolbox was tested for activity in media for 18 h (Figure 1C). There was an interesting interaction between 3 h and 18 h in media, where some small molecules that were not active in media at 3 h had a significant effect after 18 h (boxed in purple). Two Mn-increasers (VU0041803, VU0050661) and two Mn-decreasers (VU0047355, VU0244366) displayed this effect. Even more interesting were four small molecules that changed the direction of Mn uptake after 18 h. Decreaser VU0063088 and increasers VU0026977, VU0135086, and VU024305 had modest decreases and increases to vehicle, respectively (boxed in blue). Those that were inactive after 18 h in media were boxed in red. In total, only eight small molecules maintained their significant effect on Mn uptake in media after 3 or 18 h, not including the four that changed directionality of their effect. An additional four had activity in media at 18 h but not at 3 h.

### 2.2. Evaluation of the Selectivity of Small Molecules for Other Divalent Metals

**Figure 2:**
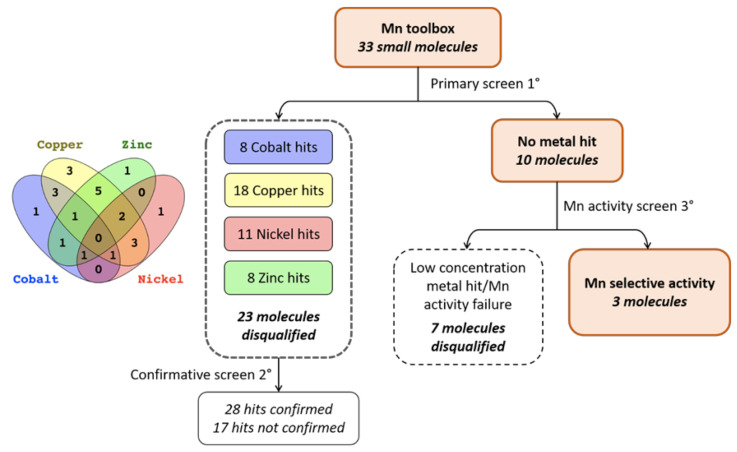
Screening paradigm for small molecule activity against other divalent metals. A primary screen of 33 small molecules was completed, assaying each molecule for fluctuations in Fura-2 fluorescence after Co, Cu, Zn, and Ni exposure (see Supplementary Table S1). Significant deviations (by one-way ANOVA, Sidak’s multiple comparisons) were detected in 23 of the small molecules, leaving 10 that were not significantly different (and within two standard deviations from the vehicle mean; see Supplementary Table S2). These 10 were tested in the presence of lower concentrations of each metal, and it was confirmed that they could indeed influence intracellular Mn levels. Three molecules remained.

Despite the fact that all of the molecules in the Mn toolbox are able to influence intracellular Mn accumulation, there was no guarantee that the small molecules were acting on mechanisms specifically regulating Mn homeostasis. Rather than perform the costly analysis of ICP-MS for multiple small molecules per divalent metal, we applied the same principle of CFMEA [34] to other divalent metals. This allowed us to screen 33 of the small molecules from the Mn toolbox against all four of these divalent metals (cobalt, copper, nickel, zinc) with the workflow displayed in Figure 2. Although 37 of the small molecules were available for testing, three molecules (VU0009101, VU0009103, VU0239513) were excluded because they were close structural homologs of other molecules in the toolbox, and another (VU0076546) was later identified to be a false positive and was subsequently removed from the Mn toolbox completely. CFMEA allows a rapid quantification of cellular Mn content at a specific time point. Following incubation with extracellular Mn, cells were washed with PBS (without Ca and Mg) to remove any remaining extracellular Mn. The cells are then lysed open with PBS + 0.1% Triton containing Fura-2, a fluorescent indicator of Mn concentration. Fura-2 fluorescence is quantitatively quenched in the presence of Mn when measured at the calcium isosbestic point, 360_Ex_/535_Em_.

**Figure 3:**
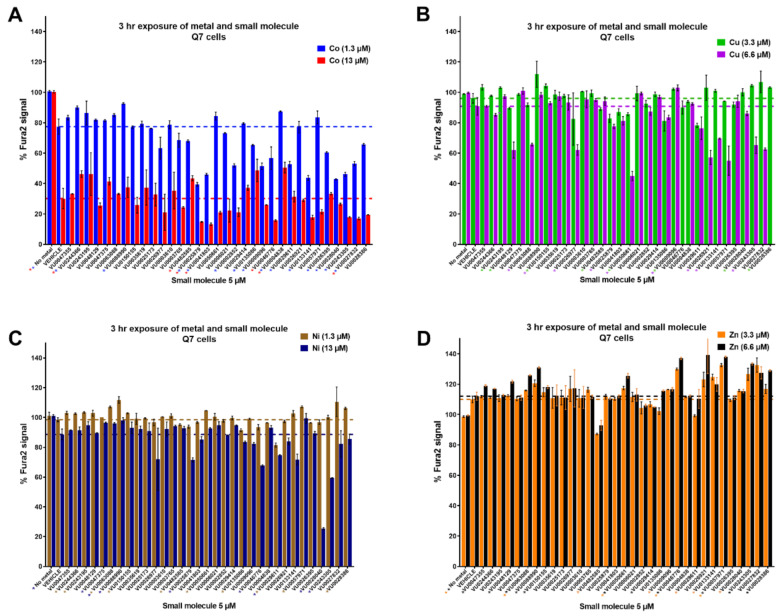
Cellular Fura-2 Divalent Metal Extraction Assay (CFXEA) screen of 33 small molecules from toolbox. Cells were pre-exposed with small molecule (5 μM), then the metal ((**A**): Cobalt, (**B**): Copper; (**C**): Nickel; (**D**): Zinc) was added. After 3 h, the cells were washed with PBS and lysed open with 0.1% Triton containing Fura-2. The presence of divalent metal quenches the Fura-2 signal in a concentration-dependent manner. We compared the Fura-2 signal of metals taken up by cells (Q7s) under vehicle conditions and determined whether the Fura-2 signal was significantly different when cells were co-exposed with a small molecule. Cells were exposed to a small molecule and metal in HBSS. The dotted line shows vehicle levels for a given metal concentration. A one-way ANOVA for each metal concentration tested was run, with post-hoc Dunnett’s T3 multiple comparisons test.

It was previously observed [35] that several other essential and divalent metals, cobalt (Co), nickel (Ni), zinc (Zn) and copper (Cu), could similarly influence Fura-2 fluorescence when added in excess. By applying the CFMEA principle to these metals, we used this newly named “Cellular Fura-2 Divalent Metal Extraction Assay” (CFXEA) test (X represents a specific divalent metal) to find small molecules that significantly increased or decreased Fura-2 fluorescence as determined by one-way ANOVA with Dunnett’s multiple comparisons test (*p* < 0.05) with a fold change greater than or less than two standard deviations from the vehicle mean. The %Fura-2 signal compared to vehicle is shown in Figure 3.

While we planned to cluster small molecules by their functionality in these CFXEA experiments (i.e., one might see patterns of similar structure/function influencing the same metals in the same direction), our primary outcome of interest was whether these small molecules were specific to Mn or if they influenced other divalent metals. Small molecules that displayed a significant influence on Fura-2 signal compared to vehicle (divalent hits) were disqualified and went through a secondary screen for confirmation of their activity (data organized in Supplementary Table S1). Molecules that did not hit any given metals were subjected to a third screen in which lower concentrations of metals were applied and their activity on Mn were confirmed (Supplementary Table S2).

**Figure 4:**
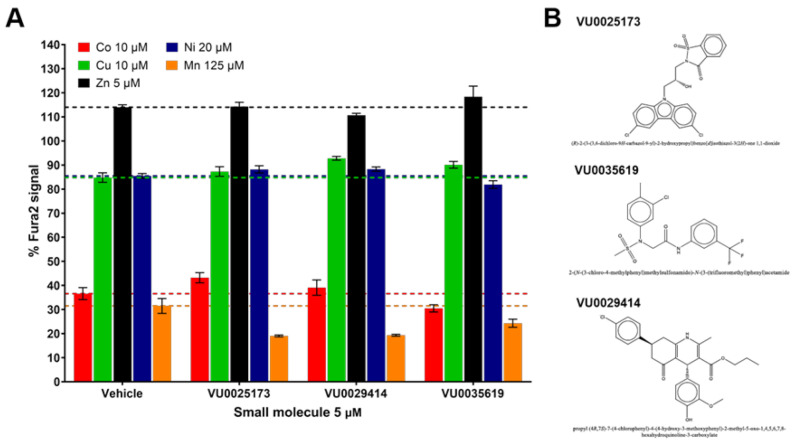
The profiles of the Mn-selective small molecules VU0025173, VU0035619, and VU0029414. Q7 cells were exposed in HBSS with DMSO vehicle or small molecules (5 μM) with a single divalent metal (Co 10 μM; Cu 10 μM; Zn 5 μM; Ni 20 μM; or Mn 125 μM) for 2 h at 37 °C. Following the exposure, cells were washed with PBS and lysed open with PBS + 0.1% Triton for CFXEA. (**A**) shows the %Fura-2 signal of CFXEA with means ± standard deviations. For ease of visualization a colored dashed line is shown at the %Fura-2 signal of the vehicle control for each metal. (**B**) shows the chemical structures of the molecules.

This left us with just three small molecules (VU0035619, VU0025173, and VU0029414), highlighted in green in Supplementary Table S2, that influenced Mn but not Co, Cu, Zn, or Ni. They were all associated with relatively small Mn increasers (~1.5 fold). Two small molecules (VU0003610 and VU0003765) did not confirm Mn activity. A replicate CFXEA was performed where the activities of the three selected molecules were confirmed at higher extracellular concentrations (Figure 4). As shown in Figure 4A, none of the three molecules had a significant influence on the intracellular levels of Co, Cu, Zn, or Ni. Meanwhile, all three molecules decreased %Fura-2 fluorescence signal compared to vehicle, indicating an increased cellular Mn uptake when cells were exposed to Mn with the presence of these three small molecules. The structure of these Mn-selective molecules is provided in Figure 4B. This experiment confirmed the Mn selectivity of the three small molecules determined from the divalent metal activity screen.

**Figure 5:**
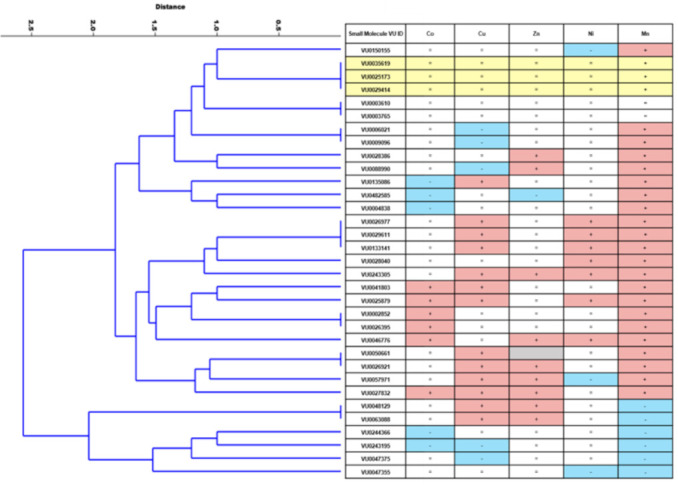
Hierarchical clustering analysis of small molecule activity for the 33 molecules screened reveals unique patterns of activity across the library. A direction (increase, decrease, no change) was assigned to each small molecule for each divalent metal based on the statistically significant results of the screening process. If no significant effect was detected, no change (marked with a “=”) was imputed. A dendrogram was then generated using a multivariate classical clustering method, a paired group algorithm, and a Euclidean similarity index. Of the 33 small molecules screened, the majority of molecules did not cluster together, demonstrating 24 distinct metal activity profiles. The assigned functional activity is listed in the table at the right of the dendrogramF and was based on the deductive logic described in the flow chart of Figure 2. Due to conflicting results in the screens, the directionality of one small molecule (VU0050661) could not be determined for Zn (marked in grey). Metal “increasers” are denoted with a “+” in a red square. Metal “decreasers” are denoted with a “-” in blue squares.

We performed a hierarchical clustering analysis using the activity of 33 small molecules across all five divalent metals screened (Co, Zn, Ni, Cu, Mn) to explore the relationship across activity type. This is depicted in the dendrogram of Figure 5. The number of terminal branches at the bottom (right) of the tree reflects how diverse the set of small molecules are in their functional specificity. For example, other than the three Mn-selective small molecules that clustered together (VU0035619, VU0025173, and VU0029414), there was only one other instance where more than two molecules clustered together. Of the 33 molecules screened, there were 24 terminal branches in the dendrogram tree. As revealed through the screen, a summary of each molecule’s defined actions against five divalent metals can be observed in the table portion of Figure 5.

## 3. Discussion

The results shown in Figure 1 demonstrate the varying utility of the small molecules in the Mn toolbox. A quick comparison of Figure 1A to Figure 1B shows a steep decline in fold change of Mn levels. It is not surprising that many small molecules lose efficacy in media from HBSS buffer, it is likely that the molecule is simply bound to proteins in the serum. Future studies using albumin-binding assays should be carried out to confirm this possibility. Those that remained active did indeed have lower EC_50_ values, and the concentrations used between conditions were static at 10 μM.

The observation that some small molecules switched direction of activity in HBSS media versus DMEM cell culture media (Figure 1) indicates that they are capable of influencing mechanisms of Mn homeostasis, rather than relying on a chelation mechanism, for example. Such small molecules may be prioritized in future studies that assess changes to candidate proteins known to induce changes in intracellular Mn. Further, it will be important to clarify how small molecule activities are influenced by the duration of treatment, the type of extracellular media, and the concentration of extracellular Mn and other metals. Here, the concentrations of metals tested were selected based on their ability to detectably alter Fura-2 fluorescence in cell lysates, and thus are not matched to each other. In addition, to better replicate previously published data, the Mn exposure concentrations at 3 h in HBSS and media were both 125 μM. To avoid toxicity, however, the concentration of Mn used in the 18-h studies was 50 μM. While previously all of these small molecules were validated in HBSS for 37 μM and 125 μM Mn, it conceptually makes sense that some molecules may not be able to influence Mn at concentrations not relevant to physiological conditions. This may be why Mn-decreasers such as VU0047355 and VU0244366 were active at 18 h (50 μM Mn) but not at 3 h (125 μM Mn). Stability of the molecules themselves at 33 vs. 37 °C may also be a contributing factor. Thus, with more detailed stability assessment of each molecule, the longevity of the molecule stability versus longevity of a molecule’s downstream effects can be better parsed. Follow-up studies with the three Mn-selective small molecules should include efforts to find the small molecule’s protein or other biological target. Our study was limited to a neuronal murine cell line (STHdh), which was also used in our original 2014 HTS screen to generate the Mn toolbox; future studies should confirm the activity and specificity of these molecules in primary cell cultures or in vivo systems. In addition, while the CFMEA assay is validated for Mn accuracy [35], the CFXEA outcomes for individual molecules should ultimately be confirmed by other methods for determining intracellular metal levels such as inductively coupled mass spectrometry (ICP-MS) or atomic absorption spectroscopy (AAS).

The results of the screen and subsequent clustering analysis in Figure 5 provided several interesting trends. For example, other divalent metal “decreasers” were rare, except in the case of already-established Mn-decreasers. Mn-decreasers clustered together, as they tended to decrease concentrations of other divalent metals as well. However, there were seven established Mn-increasers that instead displayed a decreasing effect for other divalent metals. The ability for a small molecule to increase levels of one metal, but to decrease the levels of another, might suggest biological targets of protein antiporters, rather than acting as ionophores or chelating agents (see more on this below). Though, this could also suggest compensatory changes across metals in overall metal homeostasis. Interestingly, we found no small molecules that were universal increasers or decreasers across all metals tested. Lastly, the number of distinct metal profiles (24) that were generated from 33 small molecules indicate a large diversity of metal influencers in the Mn toolbox.

It is possible that some small molecules could alter transport of divalent metals via a chelation mechanism, and not by directly affecting cellular metal biology, especially since the concentration of the metals (Co, Cu, Zn, Ni) are similar to the concentration of small molecules tested (5 μM). Such a chelation mechanism would work by depleting the available pool of free metal ions in the media, thus decreasing metal uptake simply due to a lower concentration gradient of the chelated metal. Thus, for chelation to be the exclusive mechanism of action of a given small molecule on the metals for which it shows activity, the activity detected would have to be exclusively as a “decreaser”. Only a small minority of the small molecules were found to be exclusively “decreasers” for the metals in which they had detectable activity (Figure 5, VU0244366, VU0243195, VU0047375, and VU0047355). Though any small molecule with “decreaser” activity for a given metal could theoretically act via either chelation indirectly or by altering cellular metal transport/biology, this would need to be tested in the future. Nonetheless, the majority of molecules have at least some “increaser” activity for at least one metal; therefore, this argues in favor of most of the small molecules acting in some way upon cellular metal biology directly.

Finally, despite the fact that a novel interaction between Mn and HD was published nearly a decade ago [9], and numerous subsequent studies have noted the rescue of HD phenotypes by the reinstatement of bioavailable Mn [10,22,23,39,40], the cause of this reduced Mn bioavailability is still unknown. The results of this study provide a novel approach towards potentially identifying the culprit that causes Mn deficiency in HD. These Mn-selective molecules will also provide therapeutic options for activating these Mn-deficient mechanisms and potentially restoring disease phenotypes.

## 4. Materials and Methods

### 4.1. Cell Culture

STH*dh*^Q7/Q7^ wild type immortalized murine striatal-derived cells were obtained from Coriell Cell Repository (Camden, NJ, USA). They were plated at a density of 10,000 cells per well in 96-well plates and incubated at 33 °C with 5% CO_2_ in Dulbecco’s modified eagle medium (DMEM; high glucose Sigma-Aldrich; D6546; St. Louis, MO, USA) with 10% fetal bovine serum (FBS; Atlanta Biologicals; Flowery Branch, GA, USA), 1% penicillin/streptomycin (15140–122; Life Technologies; Carlsbad, CA, USA), 2 mM GlutaMAX (Life Technologies; Carlsbad, CA, USA), 0.5 mg/mL G418 sulfate (Life Technologies; Carlsbad, CA, USA), 1X non-essential amino acids solution (Life Technologies; Carlsbad, CA, USA), and 14 mM HEPES (Life Technologies; Carlsbad, CA, USA) for 24 h prior to the assay. Cells were dissociated using 0.05% trypsin-EDTA solution (Life Technologies; Carlsbad, CA, USA).

### 4.2. Cellular Fura-2 Manganese Extraction Assay (CFMEA)

STH*dh* cells were exposed to 125 μM Mn and incubated at 37 °C (5% CO_2_) in HBSS for 3 h. This generated “vehicle” values when Mn was co-incubated with representative amounts of DMSO (Sigma-Aldrich; D8418; St. Louis, MO, USA). Other small molecules were co-incubated with the 125 μM Mn in the HBSS to assess their impact on Mn levels. After incubation, the cells were washed three times with PBS (without Ca^2+^ and Mg^2+^) to remove the extracellular Mn. Extraction buffer (PBS containing 0.1% TritonX100 (Sigma-Aldrich; T8787; St. Louis, MO, USA) and 500 nM Fura-2) was added to lyse the cells. The fluorescence of the Fura-2 in the extraction buffer was then measured at 360_Ex_/535_Em_ so that Mn could be quantified.

### 4.3. Manganese Quantification by Fura-2

A percent max of Fura-2 fluorescence 360_Ex_/535_Em_ was calculated for each condition and the corresponding zero Mn condition was defined as 100%. Background fluorescence was subtracted from all values. The concentration of Mn (nM) was calculated with the equation: [Mn] = 1138 × ((1/%max) − 1)^0.9682^, which was generated based on a standard curve of Mn quenching the Fura-2 signal [36]. The absolute values were calculated by multiplying the concentration by volume.

### 4.4. Cellular Fura-2 Divalent Metal Extraction Assay (CFXEA)

STH*dh* cells were exposed to varying concentrations of cobalt, nickel, zinc, or copper, and incubated at 37 °C in HBSS for 2 h. This generated “vehicle” values when the divalent metal was co-incubated with representative amounts of DMSO (Sigma-Aldrich; D8418; St. Louis, MO, USA). Other small molecules were co-incubated with the divalent metals in the HBSS to assess their impact on that metal’s relative levels. After incubation, the cells were washed three times with PBS (without Ca^2+^ and Mg^2+^) to remove the extracellular metal. Extraction buffer (PBS containing 0.1% TritonX100; Sigma-Aldrich; T8787; St. Louis, MO, USA and 500 nM Fura-2) was added to lyse the cells. The fluorescence of the Fura-2 in the extraction buffer was then measured at 360_Ex_/535_Em_.

### 4.5. DNA Quantification and Normalization

Cells were lysed with 0.1% triton, and 5 μL (of 100 μL) from each well was added to 95 μL Tris-EDTA buffer (Corning; 46-009-CM; Durham, NC, USA) with 1:800 PicoGreen Reagent (final; Quant-iT PicoGreen dsDNA Assay Kit; Invitrogen; P7589; Carlsbad, CA, USA). Fluorescence of each sample well was measured at 490_Ex_/525_Em_. DNA content for each well was quantified based on an equation of linear regression generated by the fluorescence of a DNA standard curve containing equal amounts of PicoGreen reagent and 0.1% Triton. Mn concentrations were normalized to DNA levels to control for cell growth between groups and observe possible cell loss from washing steps or toxicity.

### 4.6. Statistical and Data Analysis

All analyses were made using GraphPad Prism 8 software (Graphpad Holdings, LLC, San Diego, CA, USA) with either one-way ANOVA with post-hoc Dunnett’s multiple comparisons or Sidak’s multiple comparisons test, unless otherwise stated. The dendrogram of Figure 5 was generated using Past (4.02) software and a multivariate classical clustering method with a paired group algorithm (UPGMA) and using a Euclidean similarity index. The final conclusions of activities described in the table of Figure 5 were based on the same deductions described in the flow chart of Figure 2.

## 5. Conclusions

We report here new functional characterization of a set small molecules that alter cellular Mn content in a mouse striatal cell line. This includes activity over different durations of treatment and between cell media and saline buffer. In addition, we characterized the activity of the small molecules against 4 other divalent metals. This led to a pattern of activity across 5 total metals (Mn, Co, Cu, Ni, and Zn) that allowed for functional grouping by dendrogram analysis. This work led to the identification of 3 small molecules that specifically alter Mn levels in cells but not four other divalent metals.

## Data Availability

Additional data is contained with this articles associated supplementary tables, raw data is available upon request to the senior author A.B.B.

## Supplementary Materials

The following are available online at Microsoft Excel Document with Supplementary Table S1 and Supplementary Table S2.

## Sample Availability

Samples of the small molecules, in limited quantities, are available from the authors, or alternatively can be obtained commercially. Structures for all molecules are available in our prior publication (33).

## References

[1] Balachandran R.C., Mukhopadhyay S., McBride D., Veevers J., Harrison F.E., Aschner M., Haynes E.N., Bowman A.B. (2020). Brain manganese and the balance between essential roles and neurotoxicity. J. Biol. Chem..

[2] Thompson K.J., Molina R.M., Donaghey T., Savaliya S., Schwob J.E., Brain J.D. (2010). Manganese Uptake and Distribution in the Brain after Methyl Bromide-Induced Lesions in the Olfactory Epithelia. Toxicol. Sci..

[3] Finkelstein Y., Zhang N., Fitsanakis V.A., Avison M.J., Gore J.C., Aschner M. (2008). Differential deposition of Manganese in the rat brain following subchronic exposure to manganese: A T1-weighted magnetic reso-nance imaging study. Isr. Med. Assoc. J..

[4] Pfalzer A.C., Bowman A.B. (2017). Relationships Between Essential Manganese Biology and Manganese Toxicity in Neurological Disease. Curr. Environ. Health Rep..

[5] Michalke B., Fernsebner K. (2014). New insights into manganese toxicity and speciation. J. Trace Elements Med. Biol..

[6] Tuschl K., Mills P.B., Clayton P.T. (2013). Manganese and the Brain. Int. Rev. Neurobiol..

[7] Chang Y., Jin S.-U., Kim Y., Shin K.M., Lee H.J., Kim S.H., Ahn J.-H., Park S.-J., Jeong K.S., Weon Y.C. (2013). Decreased brain volumes in manganese-exposed welders. NeuroToxicology.

[8] Guilarte T.R., Burton N.C., McGlothan J.L., Verina T., Zhou Y., Alexander M., Pham L., Griswold M., Wong D.F., Syversen T. (2008). Impairment of nigrostriatal dopamine neurotransmission by manganese is mediated by pre-synaptic mechanism(s): Implications to manganese-induced parkinsonism. J. Neurochem..

[9] Williams B.B., Li D., Wegrzynowicz M., Vadodaria B.K., Anderson J.G., Kwakye G.F., Aschner M., Erikson K.M., Bowman A.B. (2009). Disease-toxicant screen reveals a neuroprotective interaction between Huntington’s disease and manganese exposure. J. Neurochem..

[10] Bichell T.J.V., Wegrzynowicz M., Tipps K.G., Bradley E.M., Uhouse M.A., Bryan M., Horning K., Fisher N., Dudek K., Halbesma T. (2017). Reduced bioavailable manganese causes striatal urea cycle pathology in Huntington’s disease mouse model. Biochim. Biophys. Acta Mol. Basis Dis..

[11] Behrens P.F., Franz P., Woodman B., Lindenberg K.S., Landwehrmeyer G.B. (2002). Impaired glutamate transport and glutamate-glutamine cycling: Downstream effects of the Huntington mutation. Brain.

[12] Carter C. (1982). Glutamine synthetase activity in Huntington’s disease. Life Sci..

[13] Tarohda T., Yamamoto M., Amamo R. (2004). Regional distribution of manganese, iron, copper, and zinc in the rat brain during development. Anal. Bioanal. Chem..

[14] Roth J.A., Ganapathy B., Ghio A.J. (2012). Manganese-induced toxicity in normal and human B lymphocyte cell lines containing a homozygous mutation in parkin. Toxicol. Vitr..

[15] Chen M.-K., Lee J.-S., McGlothan J.L., Furukawa E., Adams R.J., Alexander M., Wong D.F., Guilarte T.R. (2006). Acute manganese administration alters dopamine transporter levels in the non-human primate striatum. NeuroToxicology.

[16] Chakraborty S., Chen P., Bornhorst J., Schwerdtle T., Schumacher F., Kleuser B., Bowman A.B., Aschner M. (2015). Loss of *pdr-1/parkin* influences Mn homeostasis through altered *ferroportin* expression in *C. elegans*. Metallomics.

[17] Mukhopadhyay S. (2018). Familial manganese-induced neurotoxicity due to mutations in SLC30A10 or SLC39A14. NeuroToxicology.

[18] Ramonet D., Podhajska A., Stafa K., Sonnay S., Trancikova A., Tsika E., Pletnikova O., Troncoso J.C., Glauser L., Moore D.J. (2011). PARK9-associated ATP13A2 localizes to intracellular acidic vesicles and regulates cation homeostasis and neuronal integrity. Hum. Mol. Genet..

[19] Chun H.S., Lee H., Son J.H. (2001). Manganese induces endoplasmic reticulum (ER) stress and activates multiple caspases in nigral dopaminergic neuronal cells, SN4741. Neurosci. Lett..

[20] Rentschler G., Covolo L., Haddad A.A., Lucchini R.G., Zoni S., Broberg K. (2012). ATP13A2 (PARK9) polymorphisms influence the neurotoxic effects of manganese. NeuroToxicology.

[21] Gorell J.M., Johnson C.C., Rybicki B.A., Peterson E.L., Kortsha G.X., Brown G.G., Richardson R.J. (1999). Occupa-tional exposure to manganese, copper, lead, iron, mercury and zinc and the risk of Parkinson’s disease. NeuroToxicology.

[22] Bryan M.R., O’Brien M.T., Nordham K.D., Rose D.I.R., Foshage A.M., Joshi P., Nitin R., A Uhouse M., Di Pardo A., Zhang Z. (2019). Acute manganese treatment restores defective autophagic cargo loading in Huntington’s disease cell lines. Hum. Mol. Genet..

[23] Bryan M.R., Nordham K.D., Rose D.I.R., Brien M.T.O., Joshi P., Foshage A.M., Gonçalves F.M., Nitin R., Uhouse M.A., Aschner M. (2020). Manganese Acts upon Insulin/IGF Receptors to Phosphorylate AKT and In-crease Glucose Uptake in Huntington’s Disease Cells. Mol. Neurobiol..

[24] Williams B.B., Kwakye G.F., Wegrzynowicz M., Li D., Aschner M., Erikson K.M., Bowman A.B. (2010). Altered Manganese Homeostasis and Manganese Toxicity in a Huntington’s Disease Striatal Cell Model Are Not Explained by Defects in the Iron Transport System. Toxicol. Sci..

[25] Burdo J.R., Menzies S.L., Simpson I.A., Garrick L.M., Garrick M.D., Dolan K.G., Haile D.J., Beard J.L., Connor J.R. (2001). Distribution of Divalent Metal Transporter 1 and Metal Transport Protein 1 in the Normal and Belgrade Rat. J. Neurosci. Res..

[26] Illing A.C., Shawki A., Cunningham C.L., MacKenzie B. (2012). Substrate Profile and Metal-ion Selectivity of Human Divalent Metal-ion Transporter-1*. J. Biol. Chem..

[27] Garrick M.D., Dolan K.G., Horbinski C., Ghio A.J., Higgins D., Porubcin M., Moore E.G., Hainsworth L.N., Umbreit J.N., Conrad M.E. (2003). DMT1: A mammalian transporter for multiple metals. BioMetals.

[28] Gunter T.E., Gerstner B., Gunter K.K., Malecki J., Gelein R., Valentine W.M., Aschner M., Yule D.I. (2013). Manganese transport via the transferrin mechanism. NeuroToxicology.

[29] Crossgrove J.S., Allen D.D., Bukaveckas B.L., Rhineheimer S.S., Yokel R.A. (2003). Manganese Distribution Across the Blood–Brain Barrier. NeuroToxicology.

[30] Chen P., Bowman A.B., Mukhopadhyay S., Aschner M. (2015). SLC30A10: A novel manganese transporter. Worm.

[31] Zaki M., Issa M., Elbendary H., El-Karaksy H., Hosny H., Ghobrial C., El Safty A., El-Hennawy A., Oraby A., Selim L. (2018). Hypermanganesemia with dystonia, polycythemia and cirrhosis in 10 patients: Six novel SLC30A10 mutations and further phenotype delineation. Clin. Genet..

[32] DeWitt M.R., Chen P., Aschner M. (2013). Manganese efflux in Parkinsonism: Insights from newly characterized SLC30A10 mutations. Biochem. Biophys. Res. Commun..

[33] Kumar K.K., Lowe J.E.W., Aboud A.A., Neely M.D., Redha R., Bauer J.A., Odak M., Weaver C.D., Meiler J., Aschner M. (2015). Cellular manganese content is developmentally regulated in human dopaminergic neurons. Sci. Rep..

[34] Kwakye G.F., Li D., Kabobel O.A., Bowman A.B. (2011). Cellular fura-2 Manganese Extraction Assay (CFMEA). Curr. Protoc. Toxicol..

[35] Kwakye G.F., Li D., Bowman A.B. (2011). Novel high-throughput assay to assess cellular manganese levels in a striatal cell line model of Huntington’s disease confirms a deficit in manganese accumulation. NeuroToxicology.

[36] Kumar K.K., Aboud A.A., Patel D.K., Aschner M., Bowman A.B. (2012). Optimization of fluorescence assay of cellular manganese status for high throughput screening. J. Biochem. Mol. Toxicol..

[37] Peres T.V., Horning K.J., Bornhorst J., Schwerdtle T., Bowman A.B., Aschner M. (2018). Small Molecule Modifiers of In Vitro Manganese Transport Alter Toxicity in vivo. Biol. Trace Elem. Res..

[38] Juttukonda L.J., Beavers W.N., Unsihuay D., Kim K., Pishchany G., Horning K.J., Weiss A., Al-Tameemi H., Boyd J.M., Sulikowski G.A. (2020). A Small-Molecule Modulator of Metal Homeostasis in Gram-Positive Pathogens. mBio.

[39] Tidball A.M., Bryan M.R., Uhouse M.A., Kumar K.K., Aboud A.A., Feist J.E., Ess K.C., Neely M.D., Aschner M., Bowman A.B. (2015). A novel manganese-dependent ATM-p53 signaling pathway is selectively impaired in patient-based neuroprogenitor and murine striatal models of Huntington’s disease. Hum. Mol. Genet..

[40] Bryan M.R., Uhouse M.A., Nordham K.D., Joshi P., Rose D.I., O’Brien M.T., Aschner M., Bowman A.B. (2018). Phosphatidylinositol 3 kinase (PI3K) modulates manganese homeostasis and manganese-induced cell signaling in a murine striatal cell line. NeuroToxicology.

